# Targeting fumarate hydratase promotes mitochondrial RNA-mediated interferon production

**DOI:** 10.1038/s41586-019-0000-0

**Published:** 2023-03-08

**Authors:** Alexander Hooftman, Christian G. Peace, Dylan G. Ryan, Emily A. Day, Ming Yang, Anne F. McGettrick, Maureen Yin, Erica N. Montano, Lihong Huo, Juliana E. Toller-Kawahisa, Vincent Zecchini, Tristram A.J. Ryan, Alfonso Bolado-Carrancio, Alva M. Casey, Hiran A. Prag, Ana S.H. Costa, Gabriela De Los Santos, Mariko Ishimori, Daniel J. Wallace, Swamy Venuturupalli, Efterpi Nikitopoulou, Norma Frizzell, Cecilia Johansson, Alexander Von Kriegsheim, Michael P. Murphy, Caroline Jefferies, Christian Frezza, Luke A.J. O’Neill

**Affiliations:** 1 School of Biochemistry and Immunology, Trinity Biomedical Sciences Institute, Trinity, College Dublin; 2 MRC Cancer Unit, University of Cambridge; 3 MRC Mitochondrial Biology Unit, University of Cambridge; 4 Faculty of Medicine, University of Cologne; 5 Department of Medicine, Division of Rheumatology, Cedars-Sinai Medical Center; 6 Department of Biomedical Sciences, Cedars-Sinai Medical Center; 7 Department of Pharmacology, Ribeirao Preto Medical School, University of Sao Paulo; 8 Edinburgh Cancer Research UK Centre, University of Edinburgh; 9 Matterworks, ^444^Somerville Ave, Somerville, MA 02143; 10 David Geffen School of Medicine at University of California Los Angeles; 11 School of Medicine Columbia, University of South Carolina; 12 National Heart and Lung Institute, Imperial College London

## Abstract

Metabolic rewiring underlies macrophage effector functions, but the mechanisms involved remain incompletely defined. Here, using unbiased metabolomics and stable isotope-assisted tracing, we show induction of an inflammatory aspartate-argininosuccinate shunt following LPS stimulation. The shunt, supported by increased ASS1 expression, also leads to increased cytosolic fumarate levels and fumarate-mediated protein succination. Pharmacologic inhibition and genetic ablation of the TCA cycle enzyme FH further elevates intracellular fumarate levels, suppresses mitochondrial respiration, and increases mitochondrial membrane potential. RNA sequencing and proteomic analysis demonstrates profound inflammatory effects resulting from FH inhibition. Of note, acute FH inhibition suppresses IL-10 expression leading to increased TNF-α secretion, an effect recapitulated by fumarate esters. Unexpectedly, FH inhibition, but not fumarate esters, also increases IFN-β production through mechanisms that are driven by mitochondrial RNA (mtRNA) release and activation of the RNA sensors TLR7 and RIG-I/MDA5. This effect is recapitulated endogenously when FH is suppressed following prolonged LPS stimulation. Furthermore, cells from SLE patients also exhibit FH suppression, indicating a potential pathogenic role for this process in human disease. We therefore identify a protective role for FH in maintaining appropriate macrophage cytokine and interferon responses.

Stimulation of macrophages with the TLR4 ligand lipopolysaccharide (LPS) induces metabolic reprogramming involving rewiring of the TCA cycle and mitochondrial respiration, facilitating cytokine production. Changes in macrophage metabolism have emerged as a major regulator of inflammation^1^–^4^. While metabolic reprogramming is crucial for macrophage activation^5^, the players involved and how they regulate cytokine production remain incompletely characterised.

## LPS stimulation drives an aspartate-argin inosuccinate shunt

**Extended Data Fig. 1:**
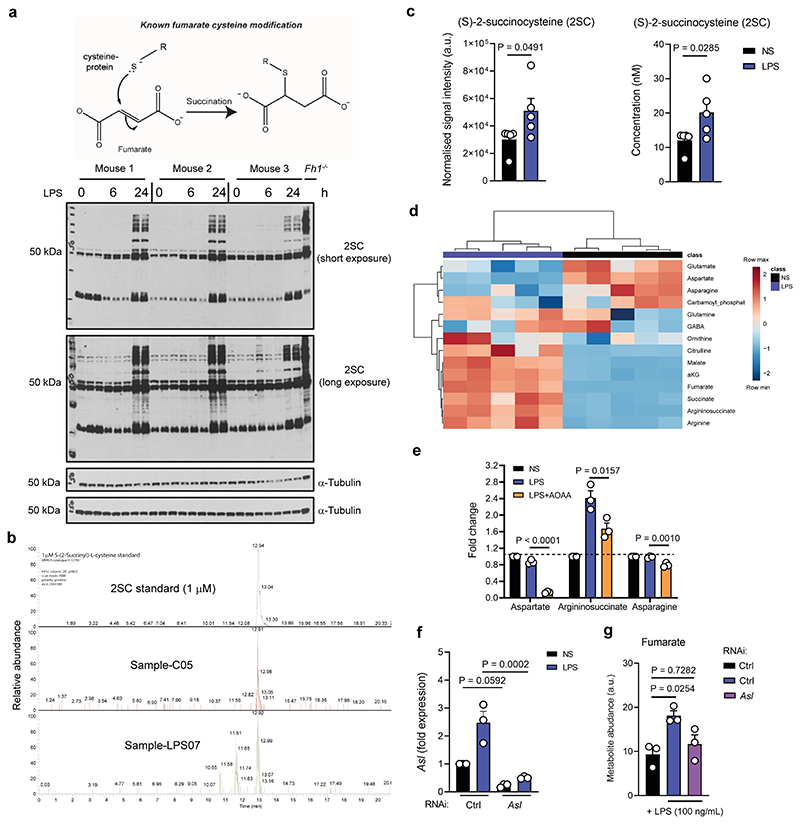
LPS stimulation drives fumarate accumulation and protein succination

**Fig. 1|:**
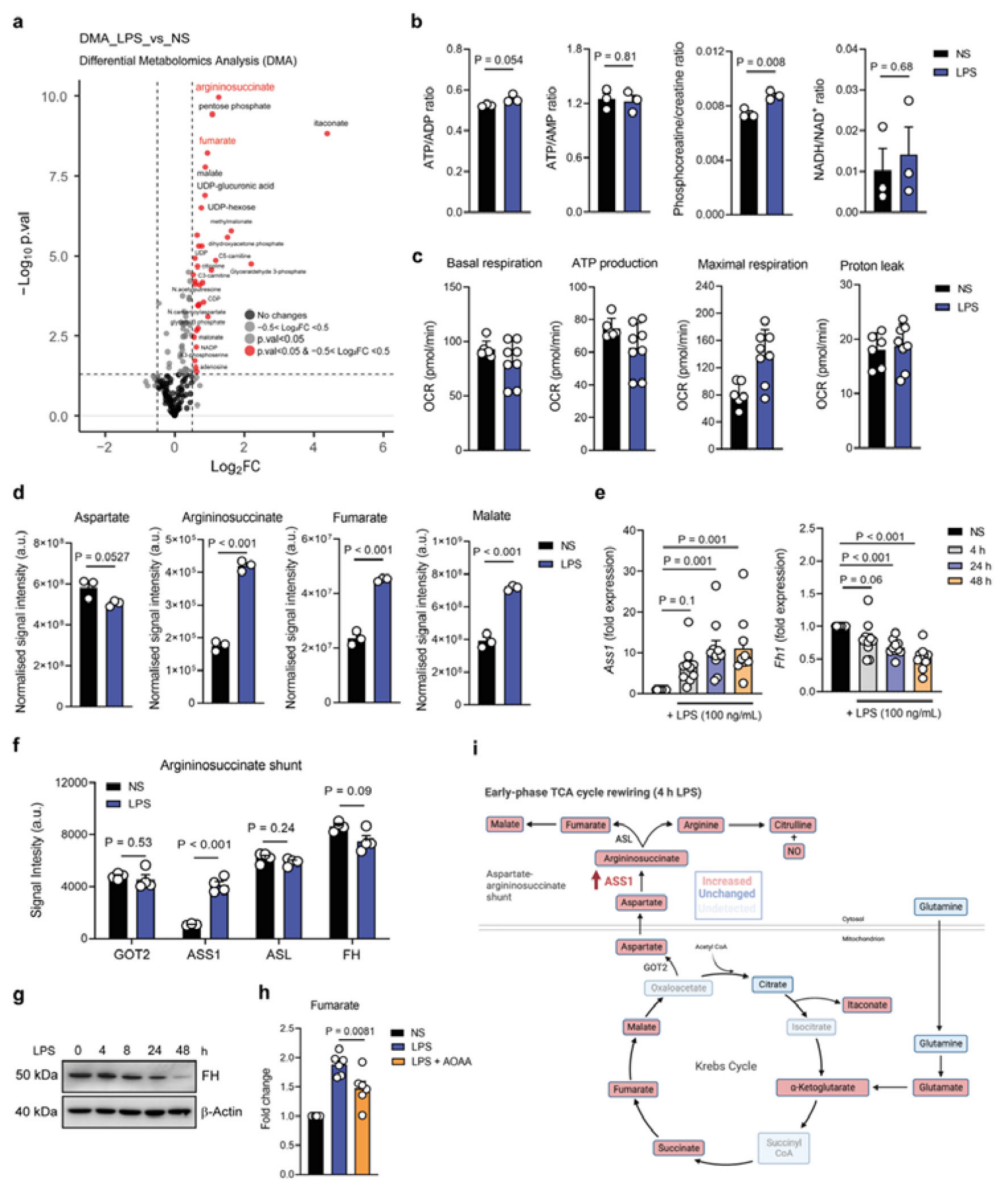
LPS stimulation drives fumarate accumulation via glutamine anaplerosis and an aspartate1225 argininosuccinate shunt Metabolite abundance (a,d) and bioenergetic ratios (b) in non-stimulated (NS) versus LPS-stimulated mouse macrophages (n =3; LPS 4 h). c, Respirometry as measured by oxygen consumption rate (OCR) of NS and LPS-stimulated mouse macrophages (n = 3; LPS 4 h). Representative experiment shown. Data are mean ± s.d. e, Ass1 and Fh1 gene expression with LPS time1229 course (n= 9). f, Quantitative proteomics of aspartate-argininosuccinate shunt enzymes in NS and LPS-stimulated mouse macrophages (n = 5, LPS 24 h). g, FH protein levels with LPS time-course (n = 1). h, Fumarate levels following LPS stimulation with or without aminooxyacetic acid (AOAA) pre-treatment (1 h) (n = 6, LPS 4 h). i, Schematic of metabolic changes occurring during early-phase TCA cycle rewiring. b,d-f,h, Data are mean ± s.e.m. g, Representative blots shown. P values calculated using two-tailed Student’s t-test for paired comparisons or one-way analysis of variance (ANOVA) for multiple comparisons.

To evaluate metabolic alterations that occur during LPS stimulation, we employed an unbiased liquid chromatography-mass spectrometry (LC-MS)-based metabolomics approach to characterise the metabolome of inflammatory bone marrow-derived macrophages (BMDMs). The TCA cycle metabolite fumarate stood out as one of the most significantly upregulated metabolites upon exposure to acute LPS stimulation, joining previously identified metabolites such as itaconate^2^ (Fig. 1a). We also observed a significant increase in fumarate-mediated protein succination^6^–^8^, resulting in the formation of the fumaratecysteine adduct, (S)-2-succinocysteine (2SC) (Extended Data Fig. 1a-c).

As acute LPS stimulation failed to impair respiration (Fig. 1b, c), TCA cycle disruption is unlikely to be sufficient for fumarate accumulation. Increased flux through the aspartate-argininosuccinate shunt has been reported to support nitric oxide (NO) production^3^. As fumarate is a by-product of argininosuccinate cleavage by argininosuccinate lyase (ASL) in the cytosol, we hypothesised that argininosuccinate may be a source of fumarate. Supporting this, we observed decreased aspartate, the substrate for argininosuccinate, and increased argininosuccinate, fumarate, and malate levels (Fig. 1d), consistent with increased flux through the shunt. This rewiring also occurred during prolonged LPS stimulation (Extended Data Fig. 1d).

Argininosuccinate synthase (Ass1) and fumarate hydratase (Fh1) expression increased and decreased respectively in LPS-stimulated BMDMs, as determined by RT-qPCR (Fig.1e). Using available quantitative proteomics data^2^,^9^, we found argininosuccinate synthase (ASS1) to be upregulated, whereas levels of glutamic-oxaloacetic transaminase 2 (GOT2), ASL and FH were not significantly altered (Fig. 1f). FH protein levels were suppressed only at later time points of LPS (Fig. 1g), indicating that ASS1 induction is vital to the acute accumulation of fumarate.

**Extended Data Fig. 2:**
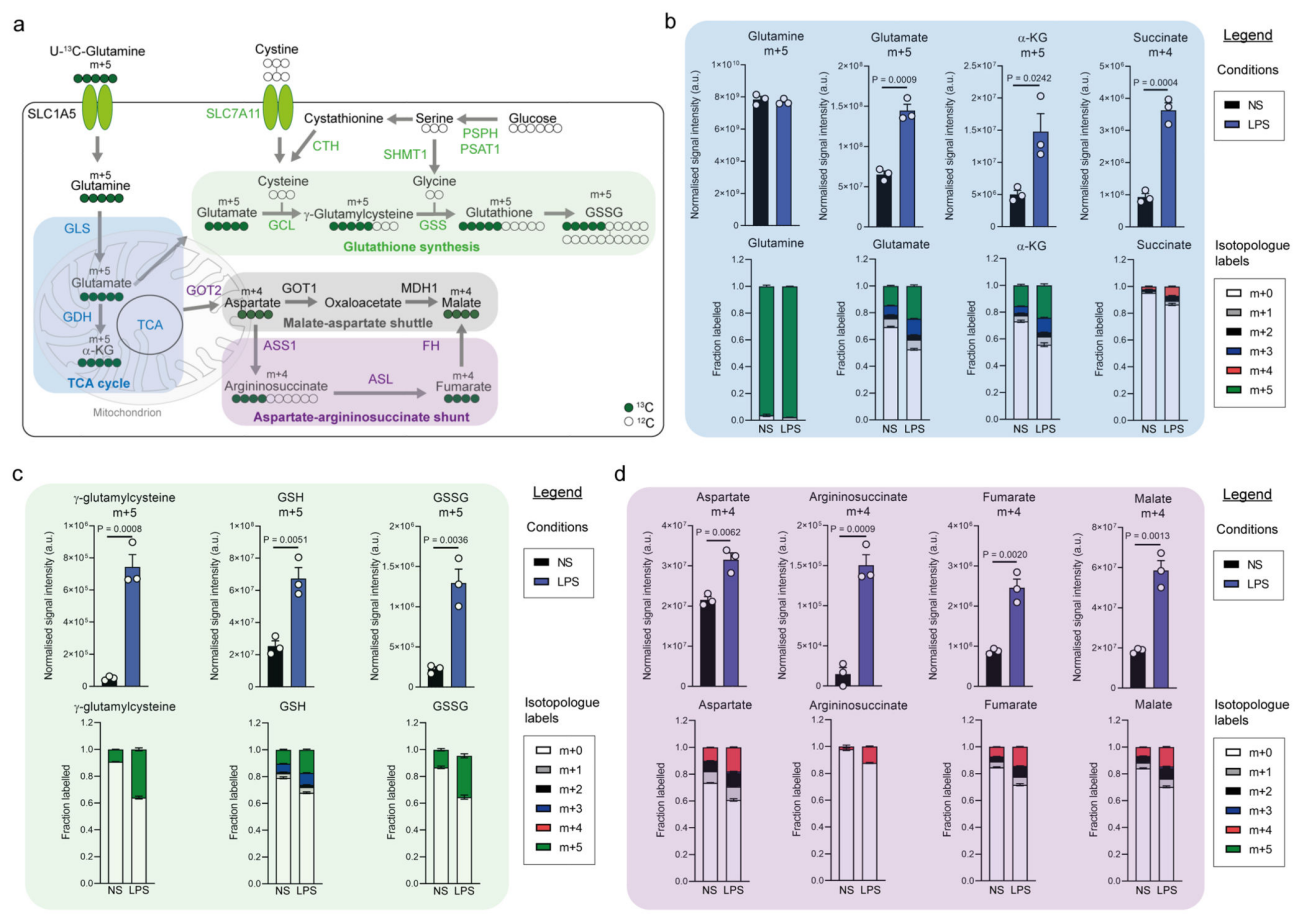
LPS stimulation drives fumarate accumulation via glutamine anaplerosis and an aspartate-argininosuccinate shunt

**Extended Data Fig. 3:**
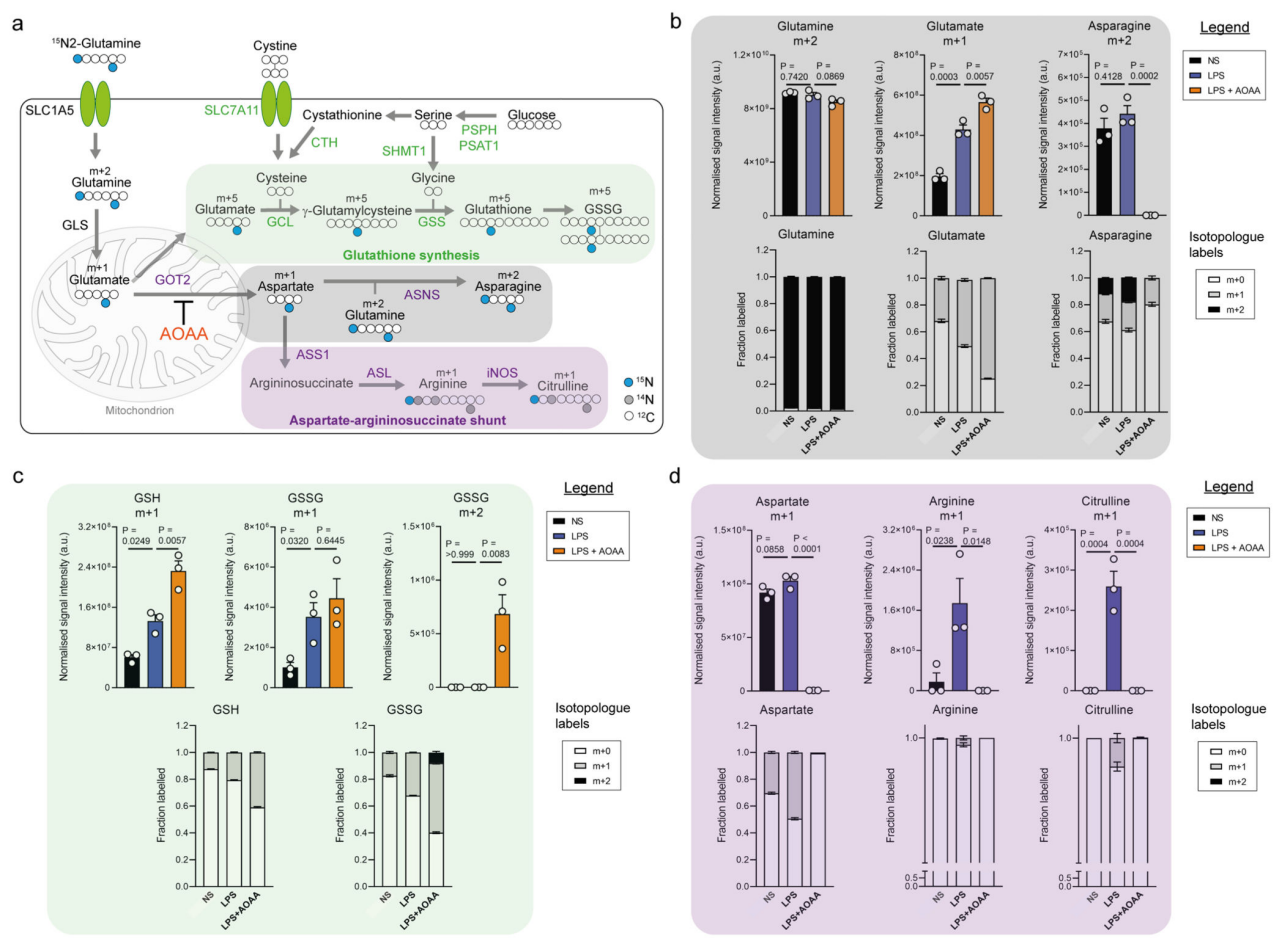
LPS stimulation drives fumarate accumulation via glutamine anaplerosis and an aspartate-argininosuccinate shunt

**Extended Data Fig. 4:**
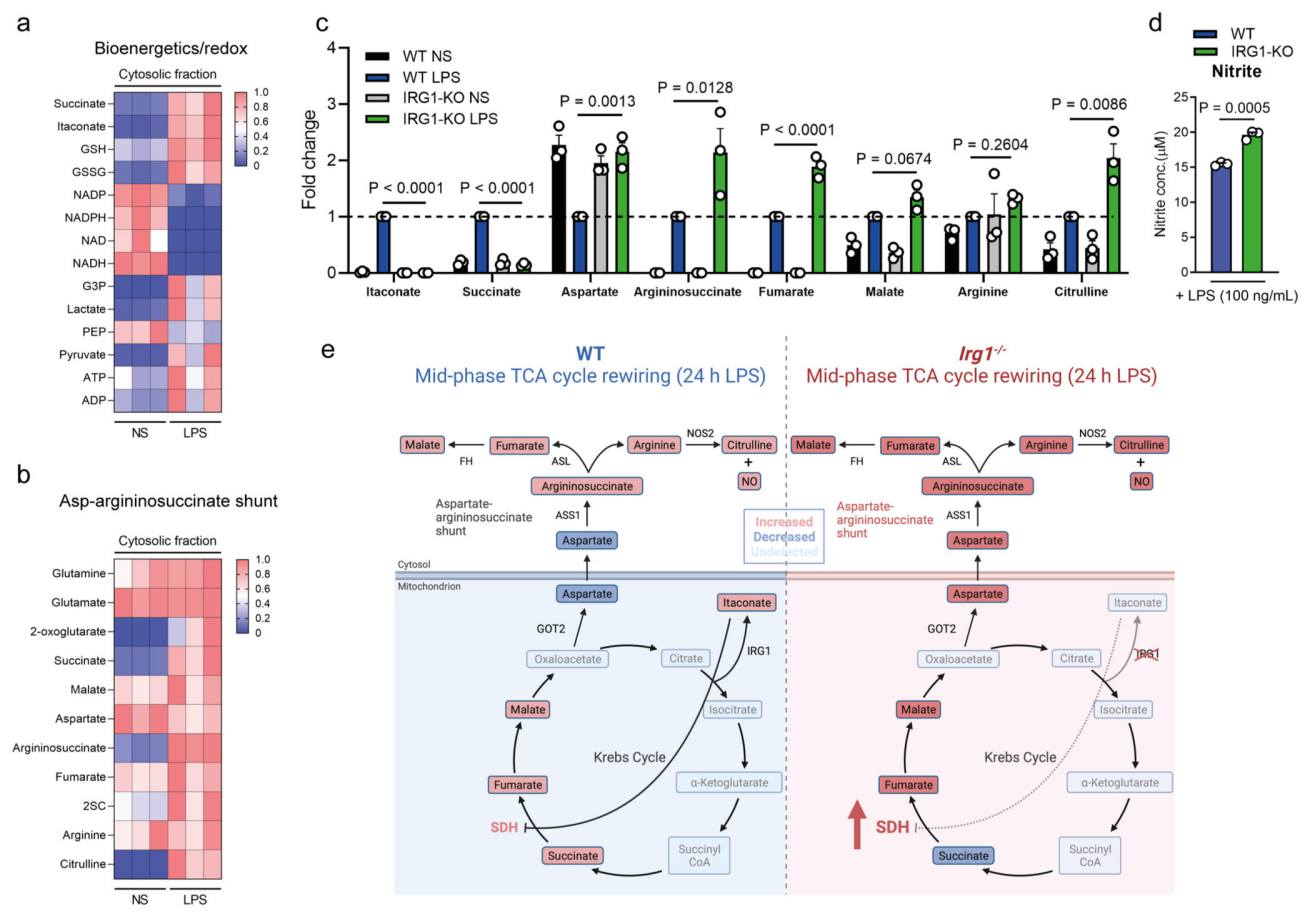
Increase in aspartate-argininosuccinate shunt metabolites in cytosol and Irg1-KO macrophages

Inhibition of the aspartate-argininosuccinate shunt with the GOT2 inhibitor aminooxyacetic acid (AOAA)3 reduced aspartate, asparagine, argininosuccinate, fumarate and 2SC levels following LPS stimulation (Fig. 1h and Extended Data Fig. 1e, f). Knockdown of Asl also prevented fumarate accumulation (Extended Data Fig. 1g, h) indicating its dependency on the aspartate-argininosuccinate shunt, which would increase cytosolic fumarate (Fig. 1i). With stable isotope-assisted tracing, we show that glutamine-dependent anaplerosis is in part responsible for fumarate accumulation and drives the aspartate-argininosuccinate shunt. U-13C-glutamine tracing demonstrated glutaminolysis as a carbon source for the TCA cycle, aspartate-argininosuccinate shunt metabolites, including fumarate, and glutathione (Extended Data Fig. 2). 15N2-glutamine tracing also demonstrated that glutamine nitrogen is a source for glutathione synthesis and aspartate-argininosuccinate shunt metabolites (Extended Data Fig. 3). Importantly, AOAA completely prevented the contribution of glutamine nitrogen to aspartate, asparagine, arginine and citrulline, confirming its inhibition of GOT2. Metabolomics on cytosolic fractions of resting and LPS-stimulated macrophages showed that metabolites such as itaconate and succinate accumulate in the cytosol following LPS stimulation (Extended Data Fig. 4a). Importantly, we also found increased cytosolic argininosuccinate, fumarate and 2SC (Extended Data Fig. 4b).

We hypothesised that Irg1-””””””””””””””””- BMDMs (which are unable to synthesise itaconate) would relieve inhibition of succinate dehydrogenase (SDH)1,10 and exhibit greater accumulation of aspartate-argininosuccinate shunt metabolites. Metabolomics in Irg1- ““““““““““““““““- BMDMs revealed the expected decrease in itaconate and succinate, and increased aspartate-argininosuccinate shunt metabolites, including fumarate and NO (Extended Data Fig. 4c, d), providing further evidence linking mitochondrial TCA cycle activity to an aspartate-argininosuccinate shunt (Extended Data Fig. 4e).

## FH inhibition causes bioenergetic and redox stress

FH catalyses the hydration of fumarate to malate in the mitochondrion and cytosol^11^, the inhibition of which elevates cytosolic fumarate accumulation, perturbs urea cycle metabolism and leads to renal cyst development^12^. Given protein levels of FH remain stable during early LPS stimulation (Fig. 1g), we used a well-established pharmacological inhibitor of FH (FHIN1)13 and a recently developed tamoxifen-inducible CRE-ERT2-Fh1-””””””””””””””””- model to probe the role of FH activity and fumarate accumulation in macrophages. However, since FH inhibition may lead to effects independent of fumarate accumulation through mitochondrial and redox stress^14^, we also used low concentrations of cell-permeable dimethyl fumarate (DMF) to deliver a cysteine-reactive fumarate ester which does not inhibit respiration^15^–^17^. This approach would uncouple the role of impaired mitochondrial bioenergetics following TCA cycle disruption and fumarate-mediated electrophilic modification of cysteine residues.

**Extended Data Fig. 5:**
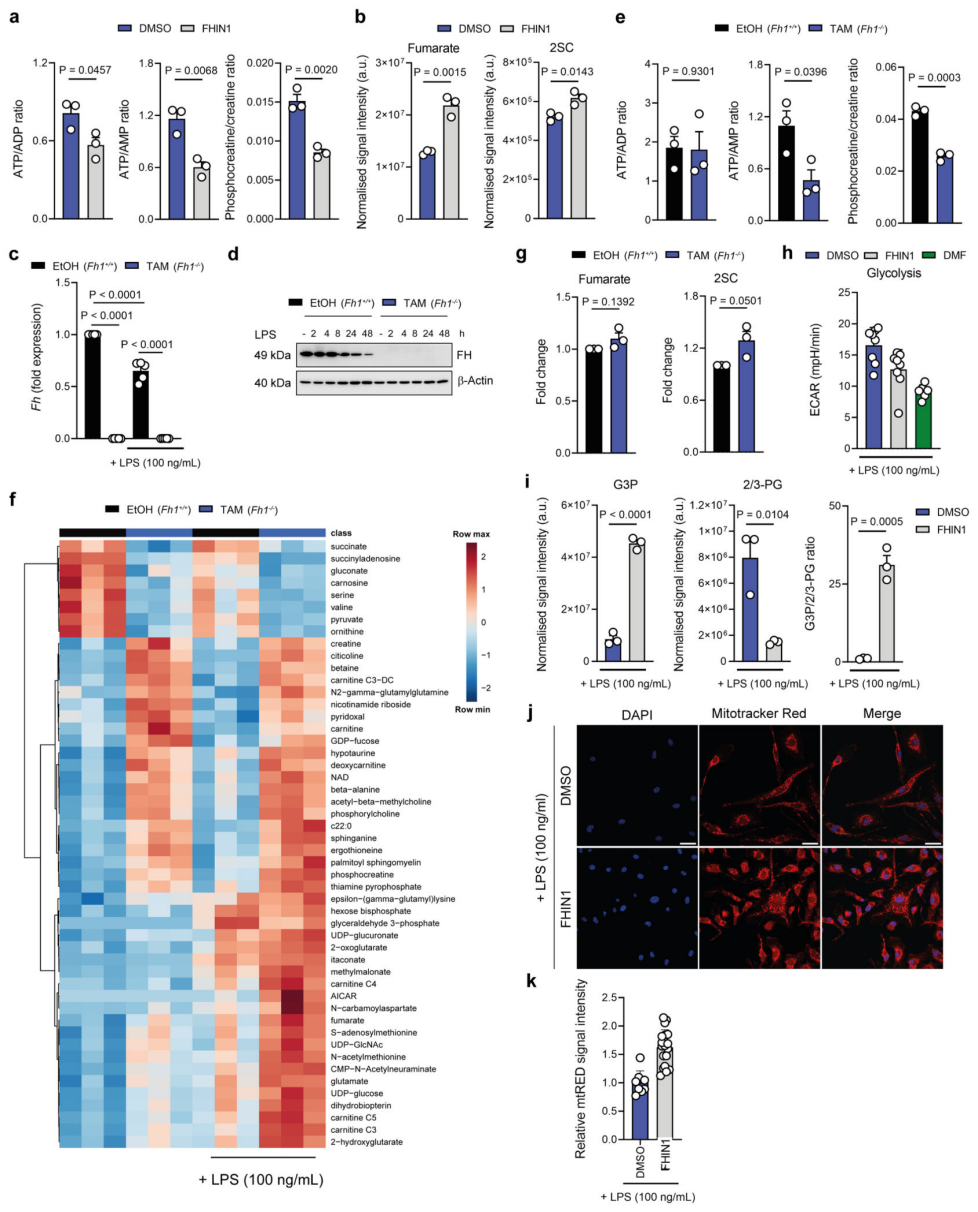
FH deletion increases bioenergetic stress, fumarate and mitochondrial membrane potential

**Fig. 2|:**
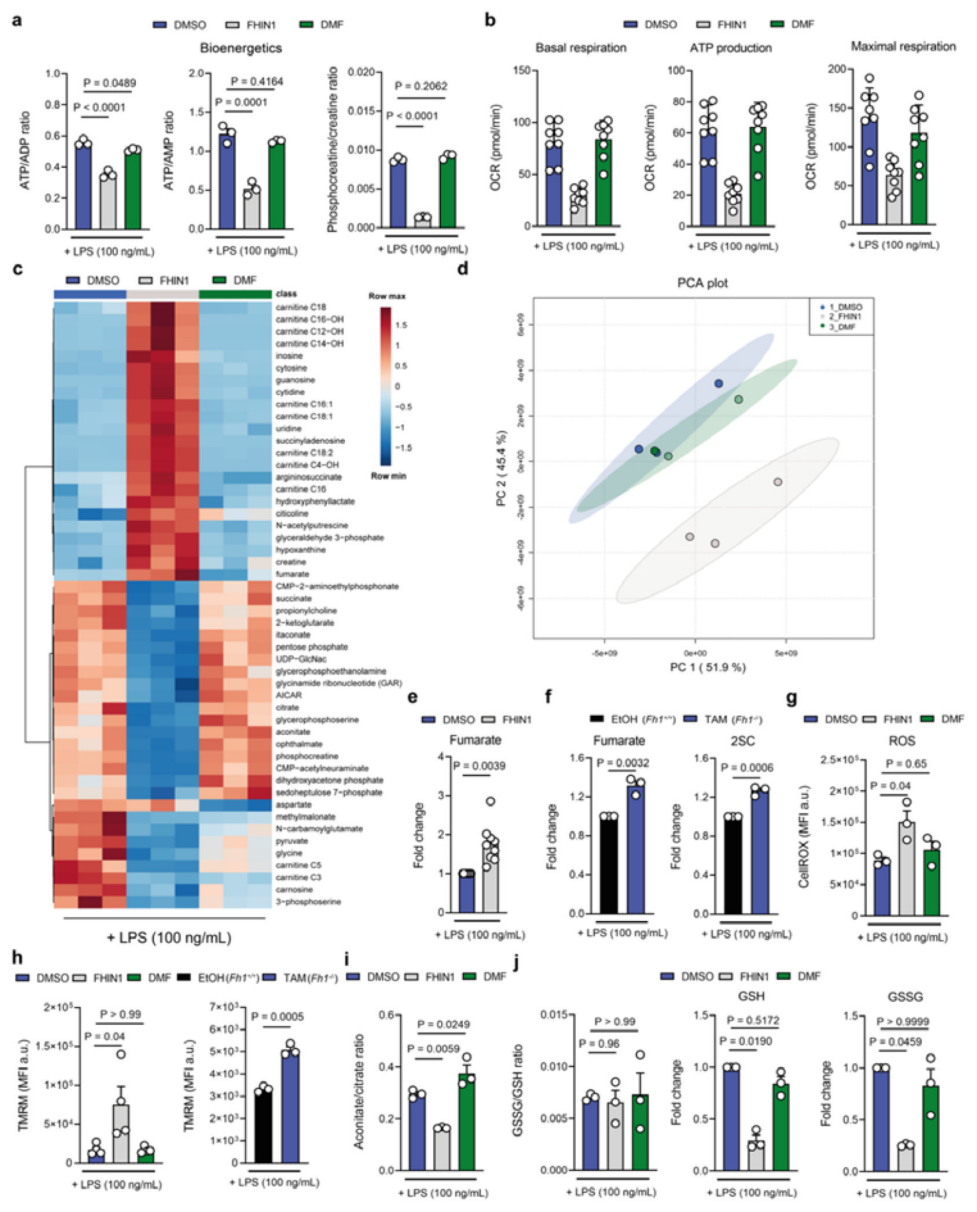
FH inhibition increases bioenergetic stress, fumarate levels and mitochondrial membrane potential Bioenergetic ratios (a) and heatmap of top 50 significantly abundant metabolites (c) in LPS-stimulated mouse macrophages pre1238 treated with vehicle (DMSO), FH inhibitor (FHIN1) or dimethyl fumarate (DMF) (n = 3; LPS 4 h). b, Respirometry of LPS-stimulated mouse macrophages pre-treated with DMSO, FHIN1 or DMF (n = 3; LPS 4 h). Representative experiment shown. Data are mean ± s.d. d, PCA plot of metabolomics of differentially abundant metabolites in LPS-stimulated mouse macrophages pre-treated (3 h) with DMSO, FHIN1 or DMF or (n = 3; LPS 4 h). e, Fumarate levels in LPS-stimulated mouse macrophages pre-treated (3 h) with DMSO or FHIN1 (n = 9; LPS 4 h). f, Fumarate and 2SC levels in LPS-stimulated Fh1+/+ and Fh1 1242 -/- mouse macrophages (n=3; LPS 4 h). g, Mean fluorescence intensity (MFI) of CellROX staining in LPS-stimulated mouse macrophages pre-treated (3 h) with DMSO, FHIN1 or DMF (n = 3-4; LPS 4 h). h, MFI of TMRM staining in LPS-stimulated mouse macrophages pre-treated (3h) with DMSO, FHIN1 or DMF or LPS-stimulated Fh1+/+ and Fh1 1245 -/- mouse macrophages (n = 3-4; LPS 4 h). i, Aconitate/citrate ratio following LPS stimulation with or without FHIN1 or DMF pretreatment (3 h) (n = 3; LPS 4 h). j, GSH and GSSG levels following LPS stimulation with or without FHIN1 or DMF pre-treatment (3 h) (n = 3; LPS 4 h). a,e-j Data are mean ± s.e.m. P values calculated using two-tailed Student’s t-test for paired comparisons or one-way or two-way ANOVA for multiple comparisons.

Previous reports show that immunometabolites and their derivatives affect macrophage function through regulation of metabolic pathways^7^,^18^,^19^. We therefore aimed to assess how FH inhibition and DMF may regulate macrophage metabolism. First, comparing the effects of FHIN1 and DMF on mitochondrial bioenergetics, we found that FHIN1 reduced ratios of ATP/ADP, ATP/AMP, and P-creatine/creatine while DMF had no effect, demonstrating that FH sustains mitochondrial bioenergetics (Fig. 2a, Extended Data Fig. 5a). This was confirmed by respirometry, showing FHIN1 impaired basal respiration, ATP production and maximal respiration as measured by OCR, while DMF had no effect (Fig. 2b). FHIN1 led to a distinct metabolic signature characterised by alterations in TCA cycle metabolites including citrate, aconitate, itaconate and succinate, indicating TCA cycle rewiring, as well as enhanced fumarate and 2SC accumulation, supporting this approach in studying the roles of FH in macrophages (Fig. 2c, e, Extended Data Fig. 5b). Principal component analysis (PCA) showed a significant divergence of FHIN1 treatment to the other conditions (Fig. 2d).

Tamoxifen-inducible knockout of Fh1 in macrophages (Extended Data Fig. 5c, d) induced similar bioenergetic changes to FHIN1, demonstrated by reduced ATP/AMP and P-creatine/creatine ratios, although the ATP/ADP ratio was unchanged (Extended Data Fig. 5e). TCA cycle rewiring was also observed in Fh1-””””””””””””””””- macrophages, although to a lesser extent than with FHIN1 (Extended Data Fig. 5f). Compensatory remodelling during initial genetic inactivation of FH may buffer some of the acute changes observed with FHIN120. Importantly however, fumarate and 2SC levels were increased in Fh1- ““““““““““““““““- macrophages (Fig. 2f, Extended Data Fig. 5g), supporting our parallel use of FHIN1 and Fh1-””””””””””””””””- macrophages.

Confirming previous reports^7^, DMF, and to a lesser extent FHIN1, suppressed glycolysis (Extended Data Fig. 5h). GAPDH is reportedly inhibited by fumarate-mediated succination^7^,^21^. Consistently, FHIN1 increased the glyceraldehyde 3-phosphate (G3P)/2/3-phosphoglycerate (2/3-PG) ratio (Extended Data Fig. 5i), suggesting that endogenous fumarate accumulation may impair GAPDH activity. This provides further evidence that FH impairment leads to modulation of cytosolic processes.

As FHIN1 impaired respiration, we examined further mitochondrial parameters. We first observed increased reactive oxygen species (ROS) production in cells treated with FHIN1 but not DMF (Fig. 2g). FHIN1 treatment also increased staining intensity of the mitochondrial membrane potential (MMP)-dependent dye mitotracker RED (mtRED) (Extended Data Fig. 5j, k). Tetramethylrhodamine methyl ester (TMRM) staining confirmed this result, as FHIN1 significantly increased staining while DMF had no effect (Fig. 2h). Similarly, Fh1-/- macrophages had increased MMP, as previously reported in kidney epithelial cells^22^ (Fig. 2h). We also observed a decreased aconitate/citrate ratio in FHIN1-treated macrophages, indicative of impairment in the fumarate- and redox-sensitive TCA cycle enzyme aconitase^23^ (Fig. 2i). Although the GSSG/GSH ratio was unchanged, FHIN1 led to a depletion of total glutathione (Fig. 2j), consistent with fumarate-mediated glutathione depletion^24^,^25^. These data suggest that FH inhibition induces profound redox stress responses.

## FH activity is required to maintain appropriate cytokine responses

**Fig. 3|:**
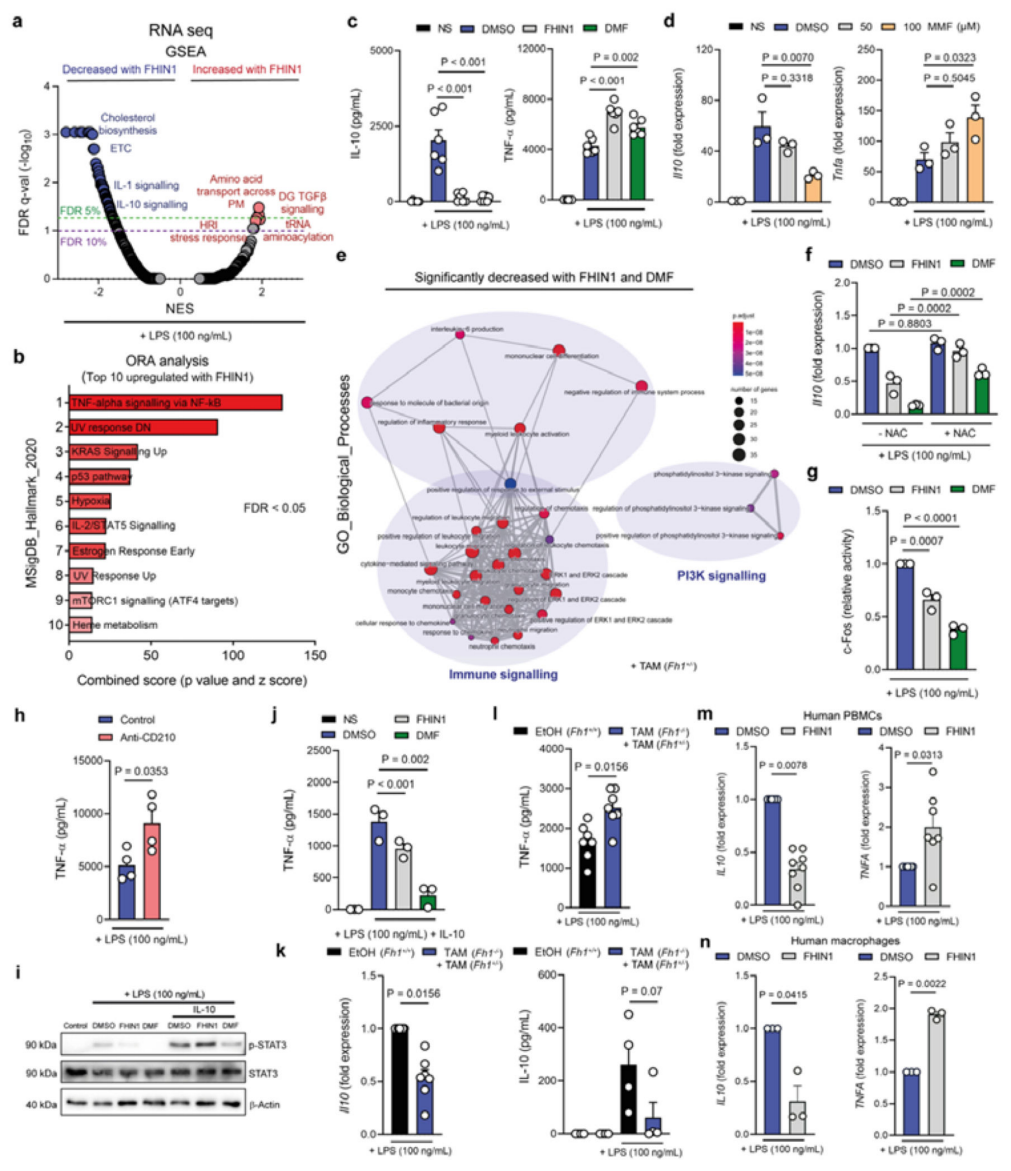
FH activity is required to maintain appropriate cytokine responses Geneset enrichment analysis (GSEA) (a) and overrepresentation analysis (ORA) (b) of significantly differentially expressed RNA seq data in LPS-stimulated mouse macrophages pre-treated (3 h) with FHIN1 compared to DMSO control (n = 3; LPS 4 h). c, ELISA of IL-10 and TNF-α in LPS-stimulated mouse macrophages pre-treated (3 h) with DMSO, FHIN1 or DMF (n = 6; LPS 4 h). d, Il10 and Tnfa expression in LPS-stimulated mouse macrophages pre-treated (3 h) with DMSO or MMF (n = 3; LPS 4 h). e, Enrichment map plot of shared significantly decreased genes in LPS-stimulated mouse macrophages pre-treated (3 h) with DMF or FHIN1 (n = 3; LPS 4 h). f, Il10 expression in LPS-stimulated macrophages pre-treated (3 h) with DMSO, FHIN1 or DMF in the presence of absence of NAC (n = 3; LPS 4 h). g, c-Fos activity in nuclear extracts of LPS-stimulated macrophages pre-treated (3 h) with DMSO, FHIN1 or DMF (n = 3; LPS 4 h). h, ELISA of TNF-α release from LPS-stimulated macrophages pre-treated with anti-CD210 antibody (1 h) (n = 4; LPS 4 h). Western blot for STAT3 and phospho-STAT3 (i) and ELISA of TNF-α release (j) from LPS-stimulated macrophages pre-treated (3 h) with FHIN1 or DMF and co-treated with recombinant IL-10 (n = 3, LPS 4 h). k, Il10 expression and ELISA of IL-10 release in LPS-stimulated Fh1+/+ and Fh1-/- (n = 5)/Fh1+/- 1262 (n = 2) macrophages (LPS 4 h). l, ELISA of TNF-α in LPS-stimulated Fh1+/+ and Fh1-/- (n = 5)/Fh1+/- 1263 (n = 2) macrophages (LPS 4 h). m, IL10 and TNFA expression in LPS-stimulated human PBMCs pre-treated (3 h) with DMSO or FHIN1 (n = 8, LPS 4 h). n, IL10 and TNFA expression in LPS1265 stimulated human macrophages pre-treated (3 h) with DMSO or FHIN1 (n=3, LPS 4 h). c,d,f-h,j-n Data are mean ± s.e.m. i, Representative blots shown. P values calculated using two-tailed Student’s t-test for paired comparisons or one-way ANOVA for multiple comparisons.

To determine whether FH regulates macrophage activation and effector responses, we performed RNA sequencing and proteomics to assess changes in the transcriptome and proteome of FHIN1-treated BMDMs. Geneset enrichment analysis (GSEA) identified an expected suppression in genes associated with metabolism, but FHIN1 also decreased expression of inflammatory pathways, including IL-1 and IL-10 signalling (Fig. 3a). Increased expression of the heme-regulated inhibitor (HRI) stress response, amino acid metabolism and tRNA aminoacylation was also observed (Fig. 3a), consistent with previous reports^14^. Further overrepresentation analysis (ORA) of RNAseq data revealed TNF-α signalling to be the most highly upregulated pathway in our analysis (Fig. 3b).

**Extended Data Fig. 6:**
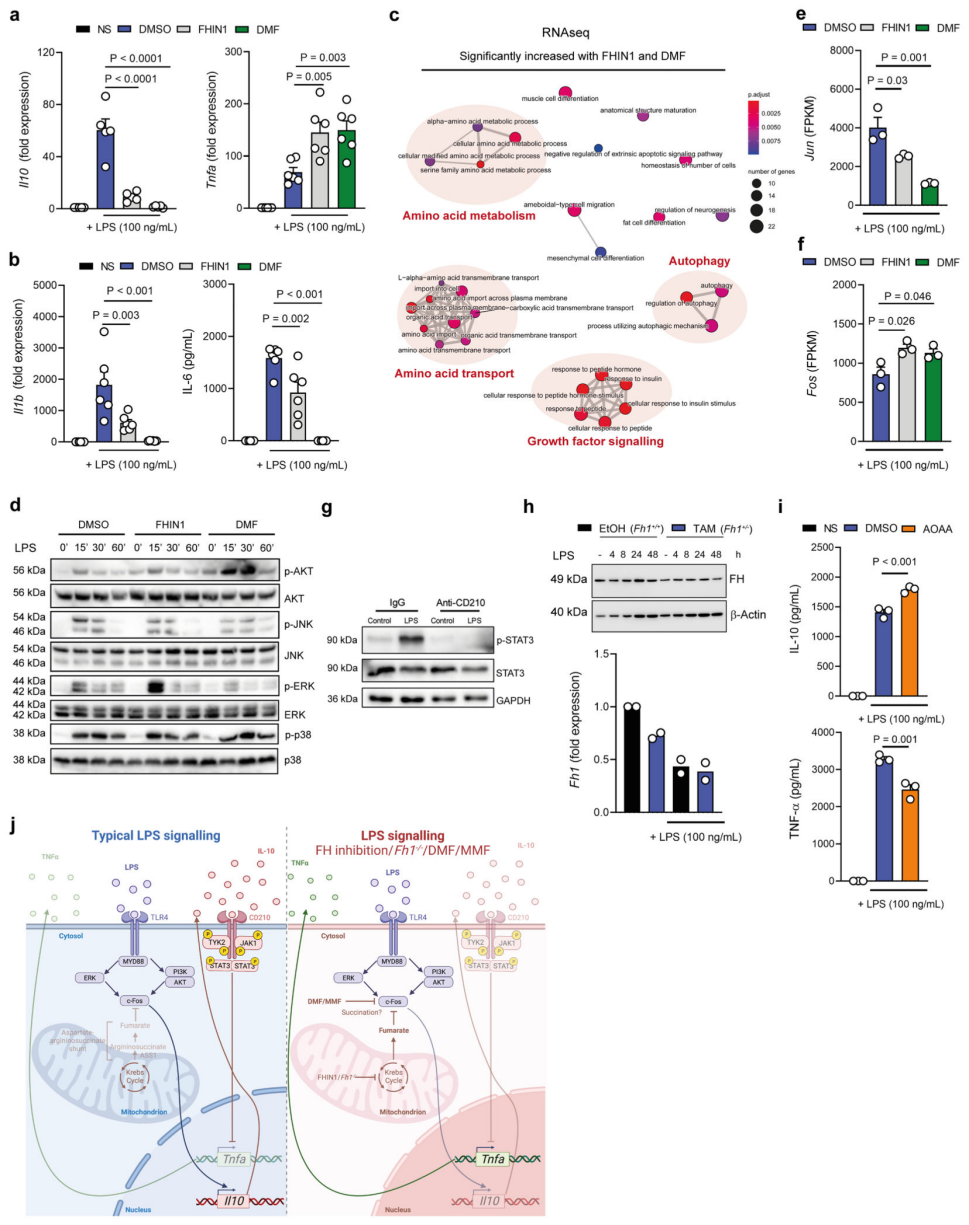
FH inhibition remodels inflammatory gene expression

Comparing FHIN1 with DMF on cytokine readouts allowed us to determine the role of protein succination following FH inhibition. Validating our transcriptomic analysis, FHIN1 and DMF decreased IL-10 release and expression, while TNF-α release and expression were increased (Fig. 3c, Extended Data Fig. 6a). Both compounds also reduced IL-1β expression and IL-6 release (Extended Data Fig. 6b), consistent with previous reports^8^,^26^, demonstrating widespread regulation of cytokine expression.

The less electrophilic fumarate ester, monomethyl fumarate (MMF), exhibited the same effects on Il10 and Tnfa expression (Fig. 3d), supporting a role for their regulation by fumarate. Shared transcriptomic changes of FHIN1 and DMF demonstrated strong downregulation of the ERK1/2 cascade and PI3K signalling (Fig. 3e). A similar transcriptional fingerprint has been observed in FH-deficient leiomyomas^27^. We also observed increased amino acid metabolism and transport, and autophagy transcripts (Extended Data Fig. 6c). Upon LPS stimulation, IL-10 is regulated by ERK1/2 and PI3K-induced AP-1 activation^28^, suggesting that downregulation of this signalling axis by FHIN1 and DMF may repress IL-10. However, we did not observe changes in the upstream kinases (AKT, JNK, ERK and p38) which converge on AP-1 activation, (Extended Data Fig. 6d). Although we did observe reduced Jun expression in our transcriptomics dataset (Extended Data Fig. 6e), this could indicate reduced autoregulation by AP-129. In this dataset, Fos was not reduced (Extended Data Fig. 6f).

Interestingly, the thiol precursor N-acetyl cysteine (NAC) abrogated the suppression of Il10 by FHIN1 and DMF (Fig. 3f). The free thiols of NAC and its products would react with and sequester fumarate, thereby reducing the modification of protein thiols and suggesting that suppression of IL-10 results from a redox-dependent succination event. The electrophile sulforaphane has been shown to reduce AP-1 activation via modification of Cys-154 on c-Fos^30^. We therefore investigated if FHIN1 or DMF may affect c-Fos activation, despite upstream regulators remaining unaffected. Using a c-Fos transcription factor assay, we found that FHIN1 and DMF strongly impaired c-Fos activation (Fig. 3g), providing evidence of direct regulation of c-Fos, potentially through S-alkylation.

IL-10 signalling has been shown to repress TNF-α expression^3^l. We confirmed this using an IL-10 receptor (CD210) blocking antibody targeting IL-10-mediated STAT3 phosphorylation, leading to augmented LPS-induced TNF-α release (Fig. 3h, Extended Data Fig. 6g). We then examined whether recombinant IL-10 supplementation could rescue the increase in TNF-α. Indeed, with IL-10, FHIN1 failed to impair STAT3 phosphorylation or augment TNF-α production (Fig. 3i, j), indicating that the FHIN1- and DMF-driven induction of TNF-α is dependent on the suppression of IL-10.

Confirming the role of FH in regulating this axis, inducible deletion of Fh1 in macrophages from heterozygous Fh1+”””””””””””””””’‘- or homozygous Fh1-””””””””””””””””- mice (Extended Data Fig. 5c, d, Extended Data Fig. 6h) resulted in decreased IL-10 expression and release (Fig. 3k) and increased TNF-α release (Fig. 3l). Furthermore, FHIN1 also suppressed IL10 expression and increased TNFA expression in LPS-stimulated human peripheral blood mononuclear cells (PBMCs) (Fig. 3m) and macrophages (Fig. 3n), indicating that the FH-regulated IL-10/TNF-α axis is also active in human cells. Establishing the role of LPS-driven fumarate accumulation on release of these cytokines, AOAA, which reduces fumarate accumulation (Fig. 1h), modestly increased and reduced IL-10 and TNF-α release respectively (Extended Data Fig. 6i), indicating that an increase in ASS1, which results in fumarate accumulation, mildly regulates IL-10 and TNF-α production. These effects are accentuated by pharmacological or genetic inhibition of FH, leading to increased fumarate accumulation (Extended Data Fig. 6j). Therefore, sustained expression and activity of FH may be viewed as protective against excessive fumarate accumulation and dysregulated production of IL-10 and TNF-α.

**Extended Data Fig. 7:**
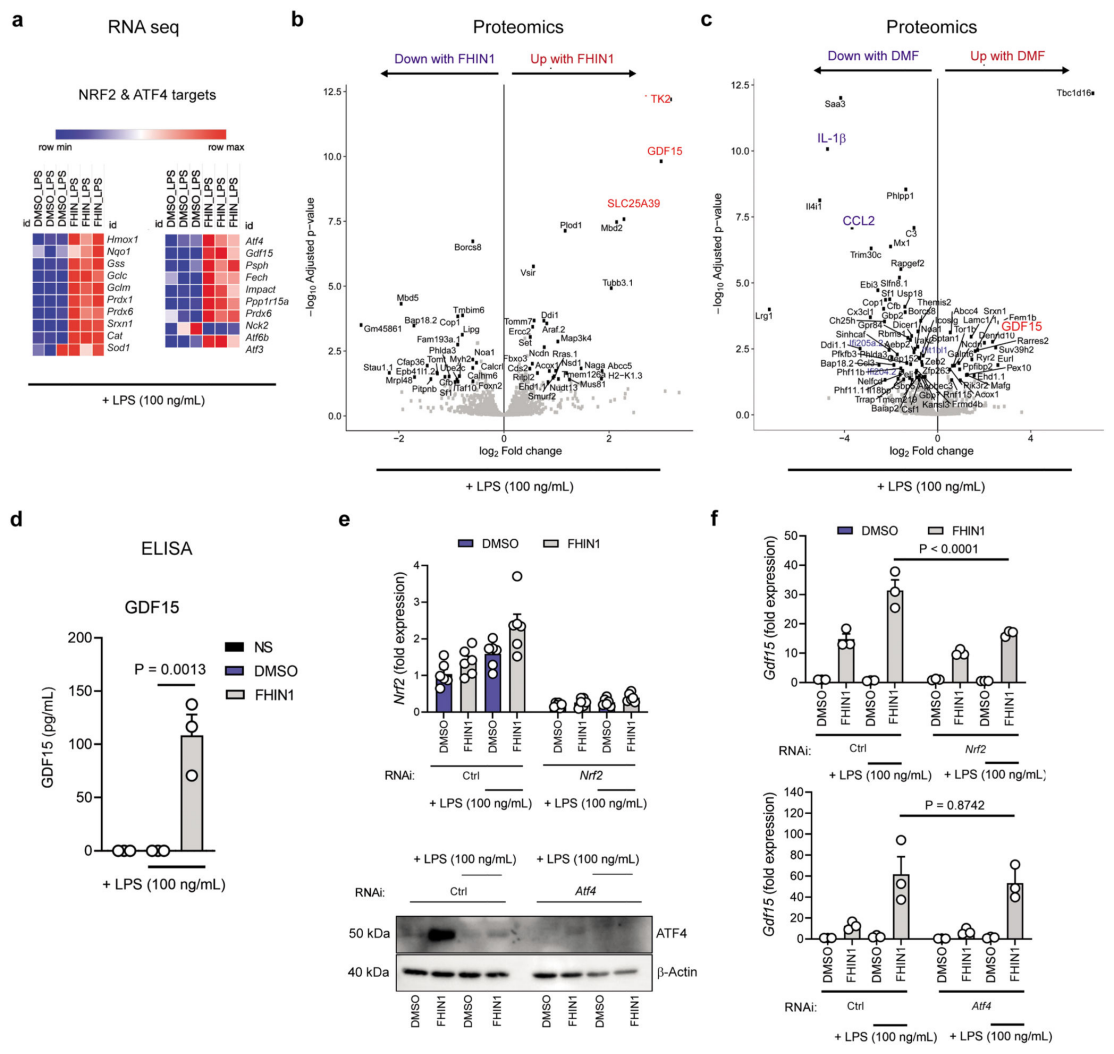
FH inhibition triggers the NRF2 and ATF4 stress response and promotes GDF15 release

FH inhibition also resulted in the activation of an NRF2 and ATF4 stress response in macrophages (Extended Data Fig. 7a), in line with previous observations in epithelial cells^14^. Proteomic analysis revealed that the inflammation-associated hormone GDF1532-34 is one of the most significantly increased proteins with FHIN1 and DMF, while FHIN1 also increased the recently identified mitochondrial glutathione importer, SLC25A3935, reinforcing the mitochondrial redox perturbation (Extended Data Fig. 7b, c). Validating our proteomics data, FH inhibition drove GDF15 release from macrophages (Extended Data Fig. 7d). Both ATF4 and NRF2 have been reported to regulate GDF15 in different contexts^33^,^36^, and silencing of each revealed that FHIN1-driven GDF15 release was partly NRF2- but not ATF4-dependent (Extended Data Fig. 7e, f). This work defines two previously unappreciated signalling axes linked to FH inhibition, uncovering its role in the regulation of IL-10/TNF-α and GDF15. The recent developments identifying GDF15 as a mediator of immune tolerance, and the anti-inflammatory properties of colchicine and NSAIDS36,37, suggest that protective effects of DMF in models of inflammation could be via GDF15. Additionally, increased TNF-α levels potentially explain adverse events reported with fumarate esters^38^. Mechanistically, suppression of IL-10 may also explain why fumarate esters promote enhanced TNF-α production during trained immunity, in addition to reported epigenetic changes^39^.

## FH inhibition triggers a mtRNA-driven retrograde type IIFN response

**Extended Data Fig. 8:**
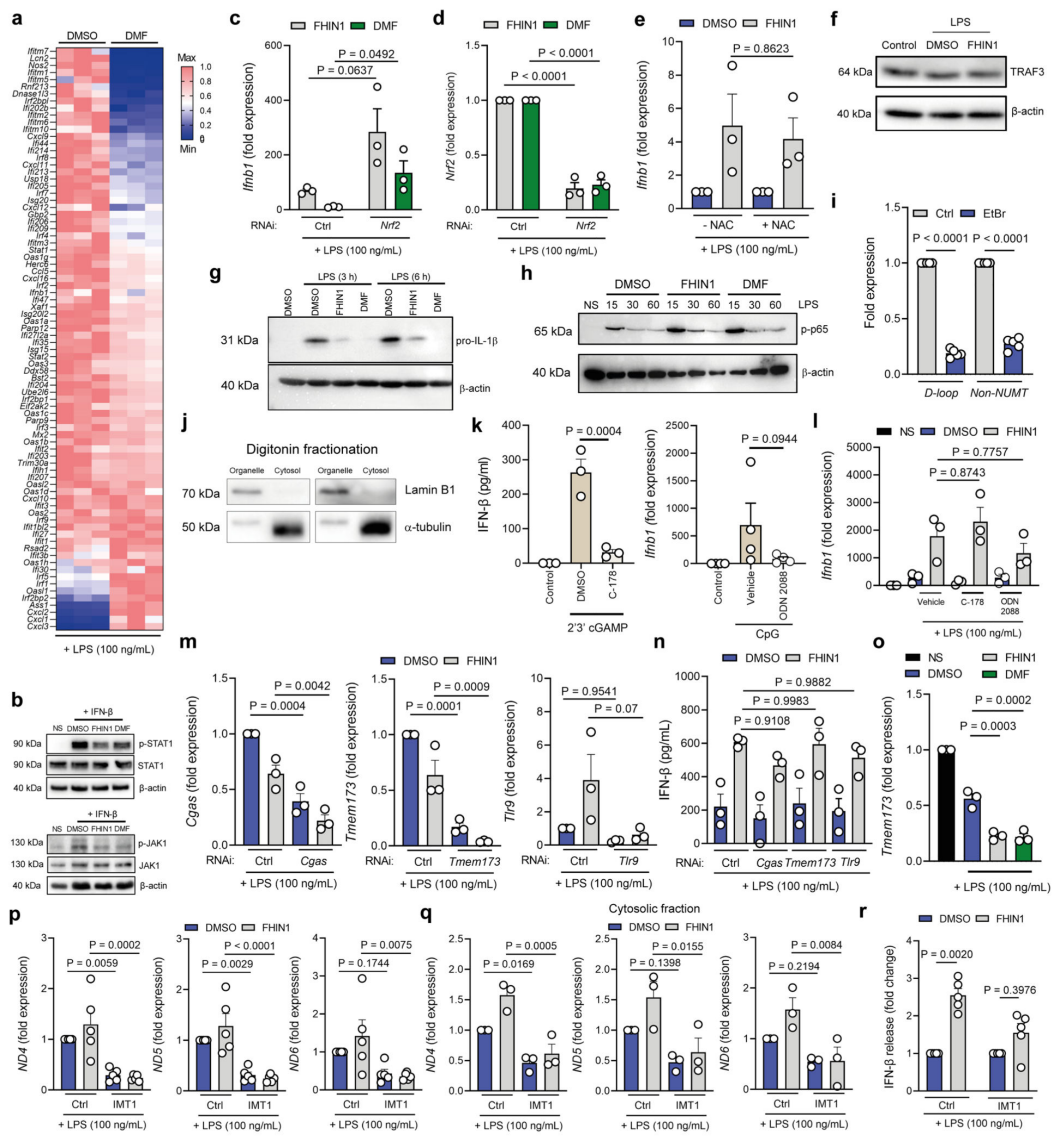
IFN-β release following FH inhibition is independent of cGAS-STING signalling

**Fig. 4|:**
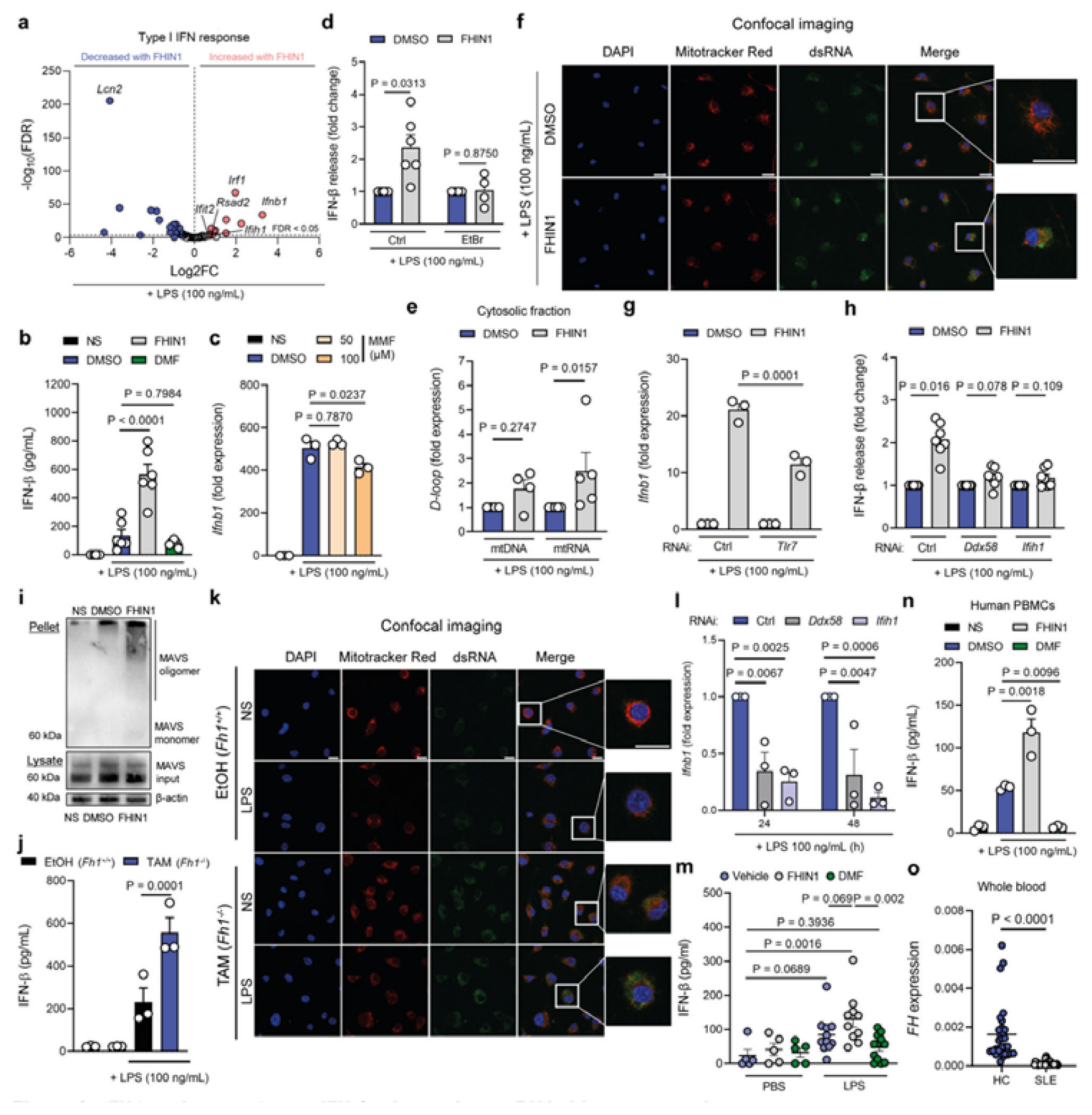
FH impairment triggers IFN-β release via a mtRNA-driven retrograde response a, GSEA of significantly differentially expressed mRNA seq data in LPS-stimulated mouse macrophages pre-treated (3 h) with FHIN1 compared to DMSO control (n = 3; LPS 4 h). b, IFN-β levels in LPS-stimulated macrophages pre-treated (3 h) with DMSO, FHIN1 or DMF (n = 6; LPS 4 h). c, Ifnb1 expression in LPS-stimulated macrophages pre-treated (3 h) with DMSO or MMF (n = 3; LPS 4 h). d, IFN-β release (fold change over DMSO control) in LPS-stimulated macrophages treated for 6 days in the presence or absence of ethidium bromide (EtBr), and subsequently pre-treated (3 h) with DMSO or FHIN1 (n = 6; LPS 4 h). e, D-loop fold expression in DNA and RNA isolated from cytosolic fractions of digitonin-fractionated LPS-stimulated mouse macrophages pre treated with DMSO or FHIN1 (n = 4 for mtDNA, n = 5 for mtRNA). f, Immunofluorescence of dsRNA in LPS-stimulated macrophages pre-treated (3 h) with DMSO or FHIN1 (n = 3; LPS 4 h). Representative experiment shown. Scale bar = 20 μM. g, Ifnb1 expression with silencing of Tlr^7^ in LPS-stimulated macrophages pre-treated (3 h) with DMSO or FHIN1 (n = 3; LPS 4 h). h, IFN-β release (fold change over DMSO control) with silencing of Ddx^58^ or Ifihl, respectively, in LPS-stimulated macrophages pre-treated (3 h) with DMSO or FHIN1 (n = 7; LPS 4 h). i, Western blot of MAVS oligomerization in LPS-stimulated macrophages pre-treated (3 h) with DMSO or FHIN1 (n = 3; LPS 4 h). j, IFN-β levels in LPS-stimulated Fh1+/+ and Fh1 l28l -/- macrophages (n = 3, LPS 4 h). k, Immunofluorescence of dsRNA in LPS-stimulated Fh1+/+ and Fh1 l282 -/macrophages (n = 3; LPS 4 h). Representative experiment shown. Scale bar = 20 μM. l, Ifnb1 expression with silencing of Ddx^58^ or Ifihl, respectively, following prolonged macrophage stimulation with LPS (n = 3). m, IFN-β levels in serum of mice injected with FHIN1 or DMF prior to injection with PBS or LPS (n = 5-11). n, IFN-β levels in LPS-stimulated human PBMCs pre-treated (3 h) with DMSO, FHIN1, or DMF (n = 3; LPS 4 h). o, FH expression in whole blood obtained from healthy controls and SLE patients (n = 30). b-e,g,h,j,i-o, Data are mean ± s.e.m. f,i,k, Representative blots or images shown. P values calculated using two-tailed Student’s t-test for paired comparisons, oneway ANOVA for multiple comparisons

RNAseq analysis of type I interferon (IFN) response genes revealed divergent effects on IFN expression and signalling with FH inhibition, including an upregulation in Ifnb1 (IFN-13) expression and several interferon-stimulated genes (ISGs), such as Irf1, Ifih1, Rsad2 and Ifit2 (Fig. 4a). However, other ISGs, such as Lcn2, were suppressed by FHIN1 and DMF treatment (Fig. 4a & Extended Data Fig. 8a). Examination of specific type I IFN signalling components downstream of the interferon-α/13 receptor (IFNAR) revealed that both FHIN1 and DMF treatment limited IFN-13-induced signal transducer and activator of transcription 1 (STAT1) and Janus kinase 1 (JAK1) phosphorylation (Extended Data Fig. 8b), indicating modest suppression of JAK/STAT signalling. Activation of NRF2 by fumarate and derivatives (Extended Data Fig. 7) may be responsible^40^. Indeed, Ifnb1 expression was increased with FHIN1 and DMF following Nrf2 silencing (Extended Data Fig. 8c, d), suggesting that Nrf2 restrains interferon transcription.

Strikingly, FHIN1, but not DMF or MMF, was found to increase IFN-13 release from LPS-stimulated macrophages (Fig. 4b, c). This was independent of NAC-sensitive redox stress (Extended Data Fig. 8e), and was not due to augmented TLR4 signalling, as LPS-induced TRAF3 levels and IL-1b expression were not increased by FHIN1 (Extended Data Fig. 8f, h). FHIN1 and DMF did modestly augment LPS-induced p65 phosphorylation (Extended Data Fig. 8g), which may contribute to increased TNF-a release^41^. Given FH inhibition causes mitochondrial stress (Fig. 2) which is associated with the release of immunostimulatory mitochondrial nucleic acids^42^–^44^, we hypothesised that the IFN response was driven by cytosolic nucleic acid sensors, such as cGAS. To support this, FH deficient-hereditary leiomyomatosis and renal cell cancer (HLRCC) tumours exhibit changes in mitochondrial DNA (mtDNA)20. We first used ethidium bromide (EtBr) to deplete mtDNA45 (Extended Data Fig. 8i) before treating cells with FHIN1 and LPS. We found that FHIN1 no longer boosted LPS-induced IFN-13 release in the presence of EtBr (Fig. 4d), indicating that increased IFN-13 release with FHIN1 may be mtDNA-dependent. We subsequently found that FHIN1 caused an increase in both mtDNA and mtRNA in cytosolic extracts (Fig. 4e, Extended Data Fig. 8j). Given the established role of mtDNA in driving IFN responses^42^,^43^, we examined whether the cGAS-STING or TLR9 DNA-sensing pathways were required for the increase in IFN-13. However, neither use of the STING inhibitor C-17846 nor silencing of Cgas (cGAS) or Tmem173 (STING) had any effect on FHIN1-driven IFN-13 induction (Extended Data Fig. 8k-n). Targeting TLR9 using the competitive inhibitor ODN 208847 or using siRNA also had no effect on this response (Extended Data Fig. 8k-n). Suppression of Tmem173 expression by FHIN1 and DMF (Extended Data Fig. 8o) may explain why cGAS-STING signalling is redundant in our model, even in the presence of cytosolic mtDNA. ETC inhibition, as we observe with FHIN1 treatment, has also been shown to inhibit STING activation^48^.

**Extended Data Fig. 9:**
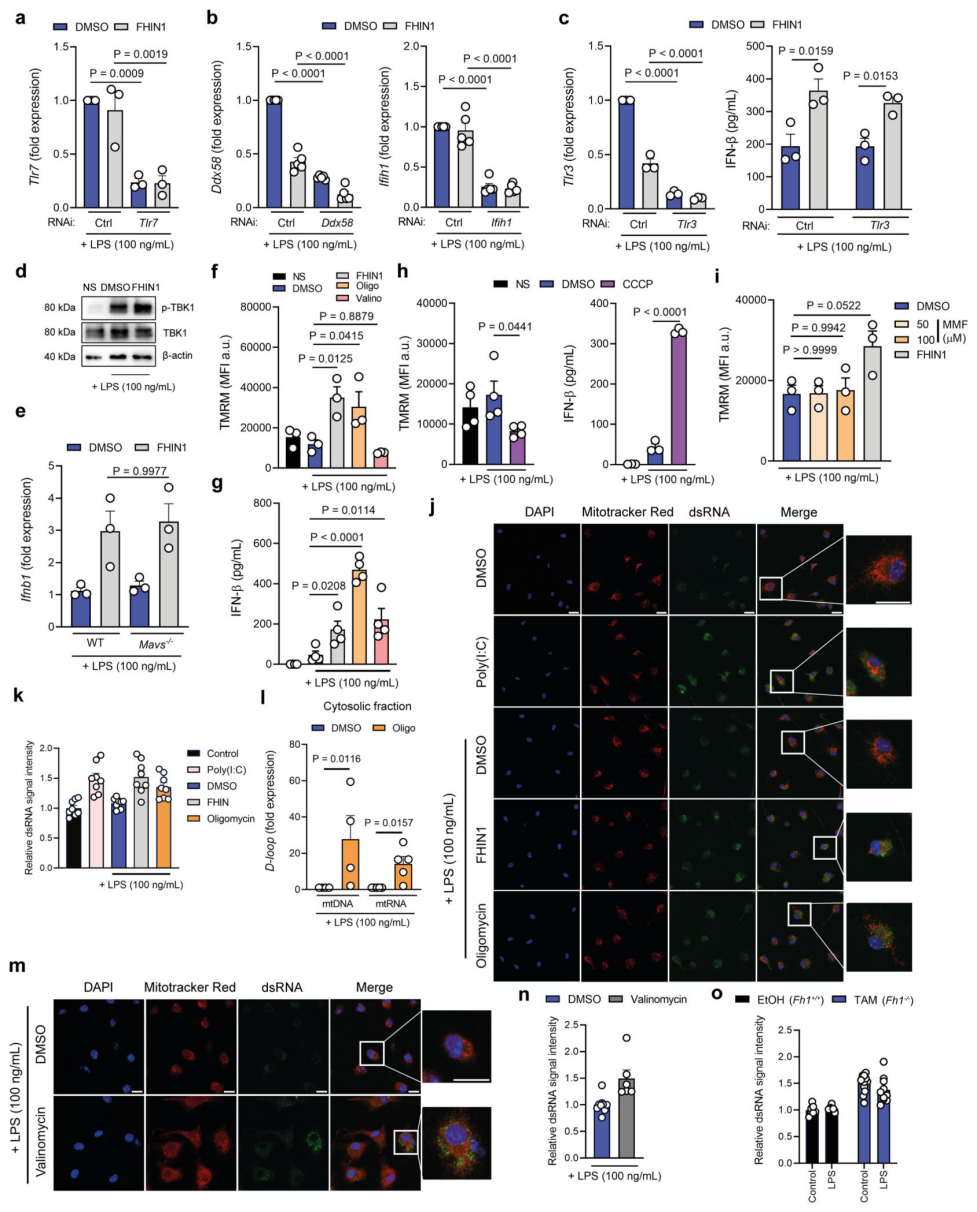
Mitochondrial membrane potential modifiers increase mtRNAand trigger IFNβ release

Since cytosolic mtRNA was also increased by FHIN1 (Fig. 4e), we performed immunofluorescence staining with an antibody specific for double-stranded RNA (dsRNA). Mitochondrial RNA has previously been shown to drive an IFN response in human cells^49^,^50^, and is known to be particularly immunostimulatory^51^. FHIN1 treatment led to an accumulation of dsRNA relative to DMSO control (Fig. 4f). We subsequently co-treated cells with FHIN1 and IMT1, the mitochondrial RNA polymerase (POLRMT) inhibitor. The increase in mtRNA with FHIN1 was observed in the cytosolic fraction but not in the whole cell fraction and was inhibited in both by co-treatment with IMT1 (Extended Data Fig. 8p, q). Importantly, IMT1 also abrogated the FHIN1-mediated boost in IFN-13 release (Extended Data Fig. 8r), implicating the role of mtRNA in driving this response. Mitochondrial ssRNA, resulting from a decline in mitochondrial integrity, has also been implicated in driving TLR7-dependent IFN signalling^52^,^53^. We subsequently silenced Tlr7 or the dsRNA sensors Ddx^58^ (RIG-I) and Ifih1 (MDA5) (Extended Data Fig. 9a, b), all of which abrogated the boost in IFN-13 release observed with FH inhibition (Fig. 4g, h), confirming a non-redundant requirement of these sensors and mtRNA, rather than mtDNA, for the FHIN1-driven IFN response. Knockdown of the cell surface dsRNA sensor Tlr^3^ did not affect the augmentation in IFN-b release (Extended Data Fig. 9c). RIG-I and MDA5, although predominantly described as dsRNA sensors, can also bind ssRNA54, indicating that the IFN response following FH inhibition is likely driven by a mixture of dsRNA and ssRNA species. It is notable that FHIN1 also reduced Ddx^58^ but not Ifih1 expression, which may warrant further investigation (Extended Data Fig. 9b). The signalling events downstream of RIG-I/MDA5 activation include mitochondrial antiviral signalling protein (MAVS) oligomerisation, followed by recruitment and phosphorylation of TANK-binding kinase 1 (TBK1). We observed MAVS oligomerisation and increased TBK1 phosphorylation with FHIN1 treatment (Fig. 4i, Extended Data Fig. 9d). Intriguingly, MAVS knockout did not impair the induction of IFN-13 by FHIN1 (Extended Data Fig.9e), perhaps indicating that compensatory TLR7 signalling is sufficient to drive type I IFN following FH inhibition with chronic MAVS deficiency.

Previously, we demonstrated that FH inhibition causes mitochondrial stress (Fig. 2). Changes in MMP have previously been correlated with increased type I IFN release^55^, thus we hypothesised that disturbances in MMP may be linked to mtRNA release and IFN-13 induction following FH inhibition. To support this, we induced changes in MMP by using the ATP synthase inhibitor oligomycin A, which boosted MMP, the K+ ionophore valinomycin A, which non-significantly reduced MMP, or the uncoupler CCCP, which significantly dissipated MMP (Extended Data Fig. 9f, h). All treatments boosted LPS-driven IFN-13 release, akin to FHIN1 (Extended Data Fig. 9g, h). MMF, which does not increase LPS-induced IFN-13 expression (Fig. 4c), did not affect MMP (Extended Data Fig. 9i). Oligomycin treatment led to an accumulation of dsRNA to a similar extent to that observed in cells treated with FHIN1 or transfected with dsRNA (poly (I:C)), and increased mtRNA release into the cytosol (Extended Data Fig. 9j-l). Valinomycin treatment similarly drove dsRNA accumulation (Extended Data Fig. 9m, n), indicating that MMP-altering compounds induce an accumulation of mtRNA. As we also observed an increase in cytosolic mtDNA levels following oligomycin treatment (Extended Data Fig. 9l), it is still possible that IFN responses following oligomycin/valinomycin/CCCP treatment are not exclusively driven by mtRNA. mtRNA release from chondrocytes has recently been implicated in activating the immune response and promoting osteo-arthritis^56^. As such, mitochondrial damage and nucleic acid release are emerging as key pathogenic processes that may underlie many immune-mediated diseases.

Tamoxifen-inducible Fh1-””””””””””””””””- BMDMs released more IFN-13 upon LPS stimulation than their Fh1+””””””””””””””””+ counterparts (Fig. 4j). We also detected increased dsRNA accumulation in Fh1-””””””””””””””””- BMDMs (Fig 4k, Extended Data Fig. 9o) which, coupled with the fact that deletion of Fh1 also drives mitochondrial membrane hyperpolarisation (Fig. 2h), demonstrate that both genetic and pharmacological targeting of FH drive similar mitochondrial retrograde type I IFN stress responses.

We next considered whether this response could be applied to an endogenous model of LPS activation in the absence of pharmacological or genetic inactivation of FH.

**Extended Data Fig. 10:**
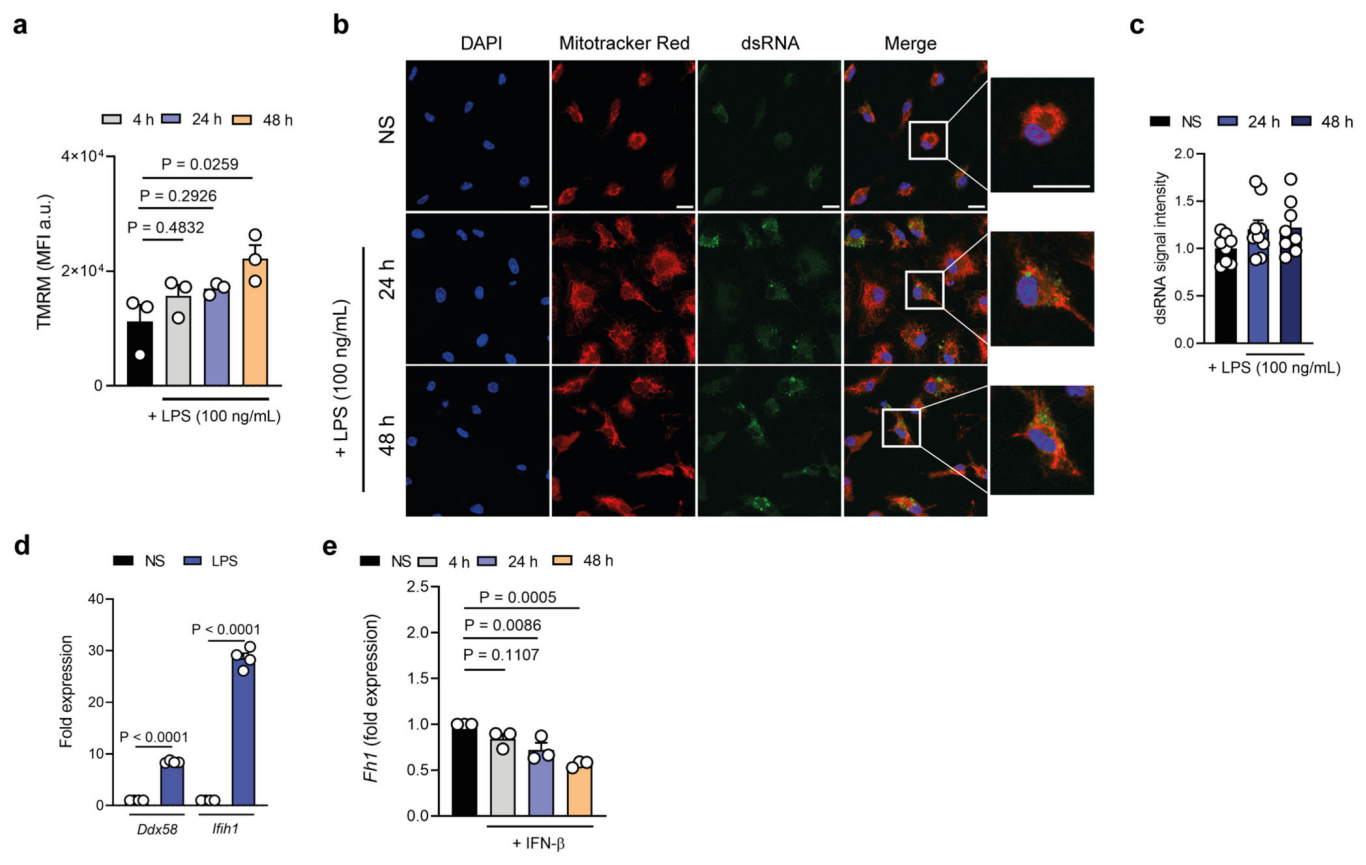
Prolonged LPS stimulation increases mitochondrial membrane potential and dsRNA

Given LPS-induced FH suppression occurs predominantly during late-phase LPS stimulation (24-48 h) (Fig. 1g), FH suppression at this time point may drive membrane hyperpolarisation and the release of mtRNA. MMP was significantly increased following 48 h LPS stimulation, but not following 4 h or 24 h stimulation (Extended Data Fig. 10a). Although dsRNA did not accumulate following acute (4 h) LPS stimulation (Extended Data Fig. 9j, k), we did observe increased dsRNA staining following 24 h and 48 h LPS stimulation (Extended Data Fig. 10b, c). Ddx^58^ and Ifihl expression is LPS-inducible (Extended Data Fig. 10d), which may suggest that RIG-I/MDA5 signalling is required during LPS stimulation. Indeed, silencing of Ddx^58^ and Ifihl reduced both 24 h and 48 h LPS-induced Ifnb1 expression (Fig. 4l), indicating that Ifnb1 transcription during late-phase LPS stimulation is maintained by mtRNA release. These results demonstrate that the mitochondrial retrograde type I IFN response, which we initially unmasked by pharmacologically or genetically targeting FH during early LPS signalling, is active endogenously during late-phase LPS activation with potential implications for chronic inflammation, for example during ageing^57^.

To determine whether FH inhibition leads to similar effects in vivo, we injected mice with FHIN1 or DMF prior to administration of LPS, and measured IFN-13 release into the serum. FHIN1 increased LPS-induced IFN-13 release, while DMF had no effect (Fig. 4m), indicating that FH inhibition leads to a similar IFN response in vivo which may have effects on bystander cells. We also treated human PBMCs with FHIN1 or DMF prior to LPS stimulation and observed similar effects, as FHIN1 boosted, while DMF suppressed LPS-induced IFN-13 release (Fig. 4n).

We hereby describe a mitochondrial retrograde signalling pathway leading from FH inhibition to mitochondrial membrane hyperpolarisation and mtRNA release (Extended Data Fig. 11). Mitochondrial stress may be an underlying mechanism that contributes to type I IFN release in interferonopathies such as systemic lupus erythematosus (SLE). It has previously been demonstrated that PBMCs from SLE patients have impaired mitochondrial function and altered MMP58,59. We therefore examined FH expression in the whole blood of SLE patients and found significant suppression of FH compared to healthy control samples (Fig. 4o). Autoantibodies to dsRNA, as well as dsDNA, have been detected in SLE patients^60^,^61^. However, it is unclear whether FHs uppression is a cause or consequence of increased IFN signalling, as Fh1 can also be inhibited by IFN-β stimulation in BMDMs (Extended Data Fig. 10e). A negative feedback loop may exist whereby suppression of FH leads to type I IFN release, which feeds back to further suppress FH. FH suppression has previously also been linked to multiple sclerosis progression^62^ and, in parallel to our work, has been shown to promote a type I IFN response in kidney epithelial cells and HLRCC tumours (Zecchini, Paupe et al., under revision). This study and ours implicate roles for FH in nucleic acid release, which may contribute to inflammation-driven tumorigenesis and as a potential host defence mechanism in the context of viral infection. Finally, the recent demonstration of aberrant dsRNA editing due to ADAR1 deficiency leading to MDA5 activation as a mechanism of common inflammatory diseases also points to the clinical relevance of endogenously produced dsRNA, suggesting that targeting this pathway may yield novel anti-inflammatory strategies^63^.

## Extended Data
